# Effects of aquatic exercise on symptoms, behaviors, and motor function in children with autism spectrum disorder: a systematic review and meta-analysis

**DOI:** 10.3389/fnbeh.2026.1927011

**Published:** 2026-08-12

**Authors:** Xianyang Xin, Yongqing Guo, Zhihao Qi, Qinzheng Liu, Longyan Liu, Xiang Ma

**Affiliations:** Capital University of Physical Education and Sports, Beijing, China

**Keywords:** aquatic exercise, autism spectrum disorder, children, meta-analysis, systematic review

## Abstract

**Background:**

Aquatic exercise combines structured physical activity, sensorimotor training, and social participation, and may be an acceptable adjunctive intervention for children with autism spectrum disorder (ASD). However, existing trials are limited by small samples, heterogeneous outcomes, and complex data structures.

**Objective:**

To evaluate the effects of aquatic exercise on ASD-related symptoms or behaviors, motor or physical function, autism severity, stereotyped behaviors, social function, and other outcomes in children with ASD.

**Methods:**

Controlled studies comparing structured aquatic exercise with passive or active controls were included. Hedges’ g was calculated using post-intervention values, with positive values favoring aquatic exercise. Random-effects models were fitted using restricted maximum likelihood with Knapp–Hartung confidence intervals. For outcomes with only 2 studies, fixed-effect sensitivity analyses were also reported. Change-score analyses and sensitivity analyses using different pre–post correlation assumptions were performed. Multiple motor outcomes from the same study were combined within study.

**Results:**

Six studies involving 159 children were included for overall ASD-related symptoms or behaviors. Aquatic exercise showed a favorable effect compared with control conditions (*g* = 1.31, 95% CI 0.63–1.99, *p* = 0.004; *I*^2^ = 45.5%), and the result remained stable in leave-one-out and change-score sensitivity analyses. Four studies involving 119 children were included for broad motor or physical function. The pooled effect favored aquatic exercise but did not reach statistical significance and showed very high heterogeneity (*g* = 2.35, 95% CI −0.11 to 4.80, *p* = 0.056; *I*^2^ = 90.2%). After excluding the study with estimated data, the effect decreased to *g* = 1.60 (95% CI −0.71 to 3.91). CARS/CARS-2, stereotyped behaviors, and social function each included only 2 studies, with unstable model-based inferences. Subgroup analyses did not identify significant differences by control type, intervention duration, or training frequency.

**Conclusion:**

Aquatic exercise may improve overall ASD-related symptoms or behaviors in children with ASD. Evidence for motor function and other specific outcomes remains limited, heterogeneous, and exploratory.

**Systematic review registration:**

https://www.crd.york.ac.uk/PROSPERO/, identifier CRD420261436475.

## 1 Introduction

Autism spectrum disorder (ASD) is a highly heterogeneous neurodevelopmental condition characterized by persistent difficulties in social communication and social interaction, as well as restricted and repetitive patterns of behavior, interests, or activities. Its manifestations extend beyond core symptoms and often require long-term, interdisciplinary, and family-centered support (Lord et al., 2018; Lai et al., 2019; Hyman et al., 2020; Lane et al., 2022; Salari et al., 2022; Zeidan et al., 2022; Hirota and King, 2023; Micai et al., 2023; Sidhu et al., 2024; Santomauro et al., 2025). In addition to core symptoms, children with ASD often present with impairments in motor coordination, balance, motor planning, and overall motor competence. These problems may limit their opportunities to participate in peer play, physical activity, and daily tasks, thereby further affecting social participation and functional development (Fournier et al., 2010; Wang et al., 2022; Kangarani-Farahani et al., 2023; Miller et al., 2023).

Structured physical activity is a non-pharmacological approach that may improve motor skills, behavioral regulation, social participation, executive function, and sleep in children with ASD (Ruggeri et al., 2019; Monteiro et al., 2022; Ji et al., 2023). However, previous reviews consistently note limitations related to small samples, heterogeneous interventions and outcomes, and variable study quality (Bremer et al., 2016; Healy et al., 2018; Huang et al., 2020; Liang et al., 2021, 2024; Wu et al., 2024; Wang et al., 2025). Proposed pathways include motor learning, sensorimotor integration, arousal regulation, social interaction, and neural or inflammation-related mechanisms, although direct mechanistic evidence remains limited (Pan et al., 2016; Toscano et al., 2021; Su et al., 2022).

Aquatic exercise is a specific form of structured physical activity. Buoyancy, resistance, hydrostatic pressure, temperature, and multisensory stimulation may reduce weight-bearing demands and support balance, coordination, body awareness, and interactive participation (Becker, 2009). Previous reviews and controlled studies have suggested potential benefits for motor, aquatic, and social-behavioral outcomes (Kumar et al., 2014; Oriel et al., 2016; Caputo et al., 2018; Mills et al., 2020; Güeita-Rodríguez et al., 2021; Naumann et al., 2021; Rohn et al., 2021; Marzouki et al., 2022; Adin and Pancar, 2023; Adibsaber et al., 2024; Kemp et al., 2024b,2024a; Ogonowska-Slodownik et al., 2024; van t Hooft et al., 2024; Zhao et al., 2024; Albadry et al., 2026; Koçak et al., 2026). However, the evidence remains limited by small samples and substantial differences in study design, intervention protocols, control conditions, staffing arrangements, and outcome measures. Because children with ASD may also have insufficient aquatic competence and an increased risk of drowning, aquatic skills and water safety remain clinically relevant (Denny et al., 2019; Kemp et al., 2024b,a).

Accordingly, the key issue is whether the reported effects of aquatic exercise are consistent across outcomes and robust to different analytical assumptions. This study therefore systematically reviewed controlled trials and quantitatively synthesized the effects of aquatic exercise on overall ASD-related symptoms or behaviors, motor or physical function, autism severity, stereotyped behaviors, and social function. Sensitivity and subgroup analyses were additionally conducted to examine the robustness of the findings.

## 2 Methods

### 2.1 Reporting guidelines and protocol registration

This study was reported in accordance with the PRISMA 2020 statement and its explanation and elaboration document (Page et al., 2021a, 2021b). The complete PRISMA 2020 checklist is provided in Supplementary Table 1. In addition, this review was registered in the International Prospective Register of Systematic Reviews (PROSPERO; registration number: CRD420261436475).

### 2.2 Search strategy and study selection

Database searches were conducted in PubMed, Web of Science Core Collection, the Cochrane Library, Embase, and Scopus, and EBSCOhost. Eligible studies were required to be available in full text, with no predefined restrictions on publication date, sample characteristics, or language. Three systematic supplementary search strategies were also used, including citation tracking and reference screening: (a) screening the reference lists of the included studies; (b) searching for subsequent studies that cited the included studies; and (c) extending the search through the “similar articles” or “find similar” functions in PubMed and Embase. The search was conducted on May 25, 2026.

The search strategy was developed based on previous systematic reviews on related topics. In addition, PROSPERO and the Cochrane Database of Systematic Reviews were searched to identify whether any relevant systematic review protocols or reviews had been published. Detailed search strategies for the other databases are provided in Supplementary Table 2.

### 2.3 Inclusion and exclusion criteria

Participants were children or adolescents with ASD diagnosed according to clinical diagnostic criteria. Studies that also included children with other neurodevelopmental disorders were eligible only when data for participants with ASD could be extracted separately. Eligible interventions were independently evaluable aquatic exercise programs, defined in this review as structured programs involving active movement performed in water. Alternative terminology was retained only in the search strategy or when reporting the terms used in the original studies. Comparators included passive or active control conditions, such as waiting-list control, no additional structured intervention, maintenance of usual activities, usual education, land-based exercise, or regular physical activity. Studies were required to report at least one outcome related to symptoms or behaviors, motor or physical function, autism severity assessed using CARS/CARS-2, stereotyped or repetitive behaviors, social function, sensory function, sleep, quality of life, executive function, or biomarkers.

Studies in which the independent effect of aquatic exercise could not be isolated were not included in the main analysis. For duplicate or overlapping trial reports, records were de-duplicated at the trial-family level to avoid repeated weighting of the same sample. For multi-arm studies, relevant aquatic intervention groups were combined, or appropriate methods were used to account for shared control groups. Crossover trials without sufficient paired data were not pooled as independent parallel-group data and were summarized narratively only. Studies that did not report precise post-intervention means and standard deviations were not converted into pseudo-precise data by visually estimating bar charts. Such studies were included in the corresponding quantitative analysis only when data were provided by the authors or could be extracted using reliable digitalization methods.

### 2.4 Data extraction and special data handling

Data extraction was independently performed by two reviewers using a prespecified Excel data extraction form (Microsoft Excel, Microsoft Corp., Redmond, WA, United States). The two reviewers independently extracted the following information: first author and publication year, study characteristics, participant demographics, training protocol, and outcome measures. When key outcome data were incompletely reported or were presented only in graphical form, the original authors were contacted to obtain the required information. If the required numerical results could not be obtained from the original authors and the relevant data were available only in graphical form, WebPlotDigitizer 4.1 was used to extract the data (Drevon et al., 2017). If the data required for a specific outcome still could not be obtained, the study was not included in the quantitative synthesis for that outcome. For each outcome in each study group, pre-intervention and post-intervention means, standard deviations (SDs), and sample sizes were extracted.

### 2.5 Risk-of-bias assessment

The risk of bias in the included randomized controlled trials was assessed using the Cochrane RoB 2 tool. The assessment was conducted for each specific outcome and covered five domains: the randomization process, deviations from intended interventions, missing outcome data, measurement of the outcome, and selection of the reported result. The risk of bias in each domain was judged as “low risk,” “some concerns,” or “high risk,” and an overall risk-of-bias judgment was then derived accordingly.

The risk-of-bias assessment was independently performed by two reviewers. During the assessment, judgments were made based on information reported in each study, including random sequence generation, allocation concealment, intervention implementation, attrition, outcome measurement methods, and selective reporting. Disagreements between the two reviewers were resolved through discussion; if necessary, a third reviewer was consulted for adjudication. The RoB 2 assessment results were presented in graphical and tabular formats.

### 2.6 Outcome definitions

The prespecified quantitative outcomes were as follows: (1) overall ASD-related symptoms or behaviors; (2) broad motor or physical function; (3) autism severity measured by CARS/CARS-2; (4) stereotyped or repetitive behaviors; and (5) social function or social impairment. Sleep, mental health, emotional regulation, sensory integration, aquatic skills, quality of life, executive function, IL-6, IL-10, and BDNF were each reported by only one independent study; therefore, conventional meta-analysis was not performed for these outcomes, and they were summarized narratively.

### 2.7 Effect size calculation and statistical analysis

The primary analysis used post-intervention values to calculate the small-sample-corrected Hedges’ g. For scales in which lower scores indicated better status, the effect direction was reversed so that g > 0 consistently indicated a beneficial effect of aquatic exercise. Post-intervention standardized mean differences and change-score effects were not combined in the same model.

When a single study reported multiple measures within the broad motor or physical function outcome domain, effect sizes were first calculated separately and then combined within the study using a generalized least squares approach. The primary analysis assumed a common within-study correlation coefficient of rho = 0.5 between outcomes, with sensitivity analyses performed using rho = 0.3 and 0.7. This approach ensured that each study contributed only one effect size to the same outcome domain.

The change-score sensitivity analysis used a Morris-type pretest-posttest-control effect size, calculated as the difference in change between the intervention and control groups divided by the pooled baseline standard deviation, with Hedges’ small-sample correction applied (Morris, 2008). When the standard deviation of change scores was not reported, it was calculated from the baseline and post-intervention standard deviations using the pre-post correlation coefficient; *r* = 0.5 was used as the primary assumption, and *r* = 0.3 and 0.7 were used in sensitivity analyses.

Pooled effects and between-study variance were estimated using REML random-effects models, and inference was conducted using the Knapp–Hartung method (Knapp and Hartung, 2003; Röver et al., 2015). For outcomes including only 2 studies, fixed-effect models were also reported as sensitivity analyses, but conclusions were not selectively drawn based on statistically significant results from either model. Heterogeneity was described using the Q test, I^2^, and tau^2^ (Higgins et al., 2003).

Leave-one-out analyses were performed for overall ASD-related symptoms or behaviors and broad motor or physical function. The prespecified subgroup variables included only comparator type, intervention duration, and training frequency. Formal tests for between-subgroup differences were conducted using Knapp–Hartung meta-regression. If any subgroup included only one study, formal between-subgroup testing was not performed.

A contour-enhanced funnel plot was generated for overall ASD-related symptoms or behaviors (Peters et al., 2008). Because only six studies were included, Egger’s test was not performed, and publication bias was not judged as present or absent based on visual inspection of the plot. All analyses were performed using R 4.4.3 and metafor 5.0-1 (Viechtbauer, 2010). Two-sided *p* < 0.05 was used as a statistical reference, but interpretation was based on effect sizes, confidence intervals, heterogeneity, the number of studies, and sensitivity analysis results.

## 3 Results

### 3.1 Study selection and study characteristics

**FIGURE 1:**
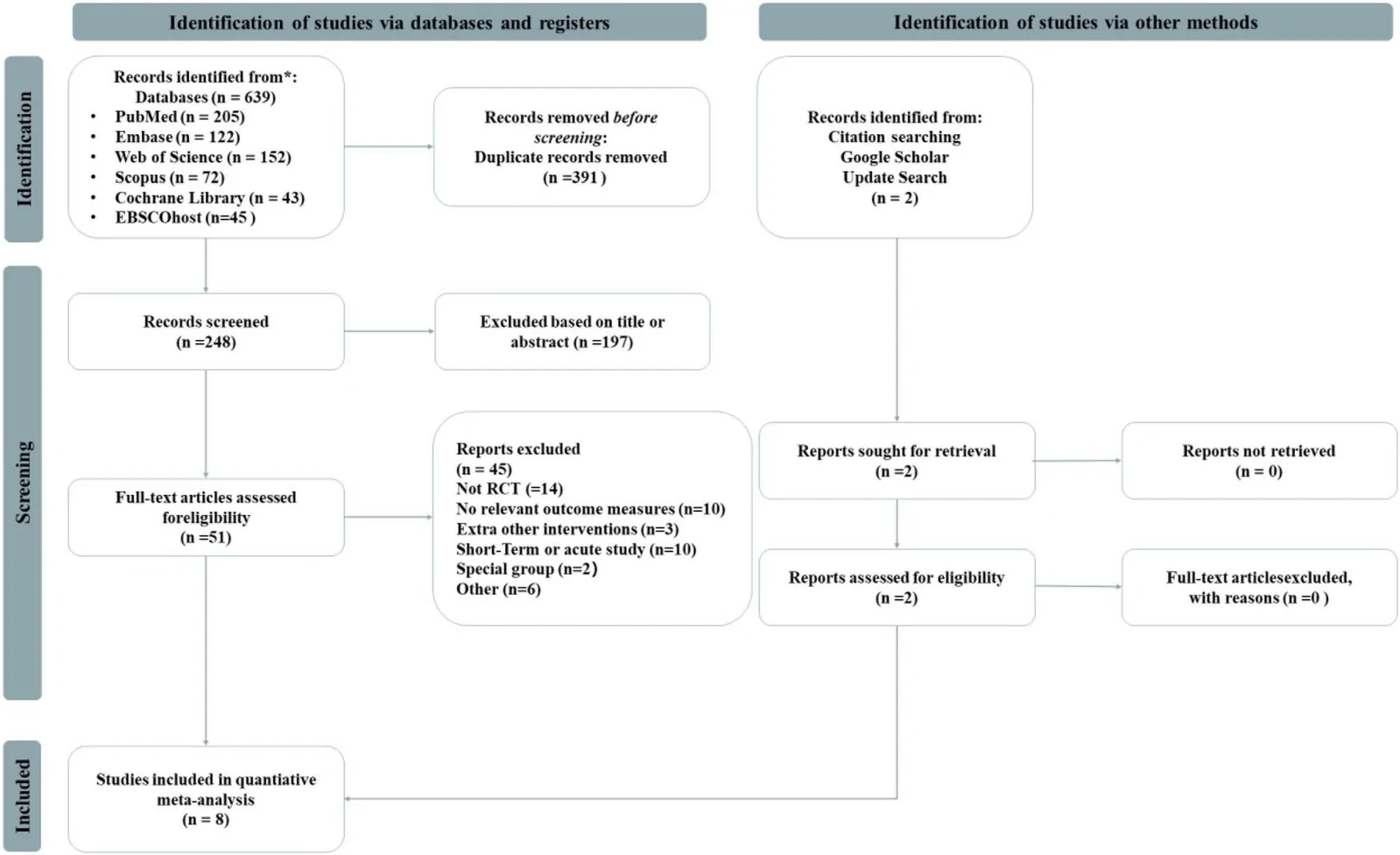
PRISMA 2020 flow diagram of study selection.

The study selection process is shown in Figure 1. A total of 639 records were identified through database searches, including 205 from PubMed, 122 from Embase, 152 from Web of Science, 72 from Scopus, 43 from the Cochrane Library, and 45 from EBSCOhost. After removing 391 duplicate records, 248 records underwent title and abstract screening, of which 197 were excluded. The remaining 51 full-text reports were assessed for eligibility, and 45 were excluded because they were not randomized controlled trials (*n* = 14), did not report relevant outcomes (*n* = 10), included additional interventions that prevented isolation of the aquatic exercise effect (*n* = 3), investigated short-term or acute effects only (*n* = 10), involved special populations (*n* = 2), or met other exclusion criteria (*n* = 6). Supplementary searches identified two additional reports, both of which were assessed for eligibility and included. Finally, eight randomized controlled trials were included in the quantitative synthesis.

The basic characteristics of the included studies are presented in Supplementary Table 3. The studies were conducted in Iran, Australia, Egypt, Turkey, Tunisia, and China, and most used randomized controlled designs. Participants were mainly boys with ASD aged 3–14 years. Aquatic exercise programs included structured aquatic training, adapted aquatic exercise, Halliwick-based programs, swimming training, and moderate-intensity aquatic exercise. Intervention duration ranged from 4 to 16 weeks, with 1–4 sessions per week and 45–70 min per session. Control conditions included waiting lists, usual activities, land-based exercise, conventional education, and unstructured land-based activities. The main outcomes included ASD-related symptoms or behaviors, motor function, stereotyped behavior, social function, sleep, quality of life, executive function, and biomarkers. The number of studies included in the quantitative synthesis varied across outcomes.

### 3.2 Risk of bias

**FIGURE 2:**
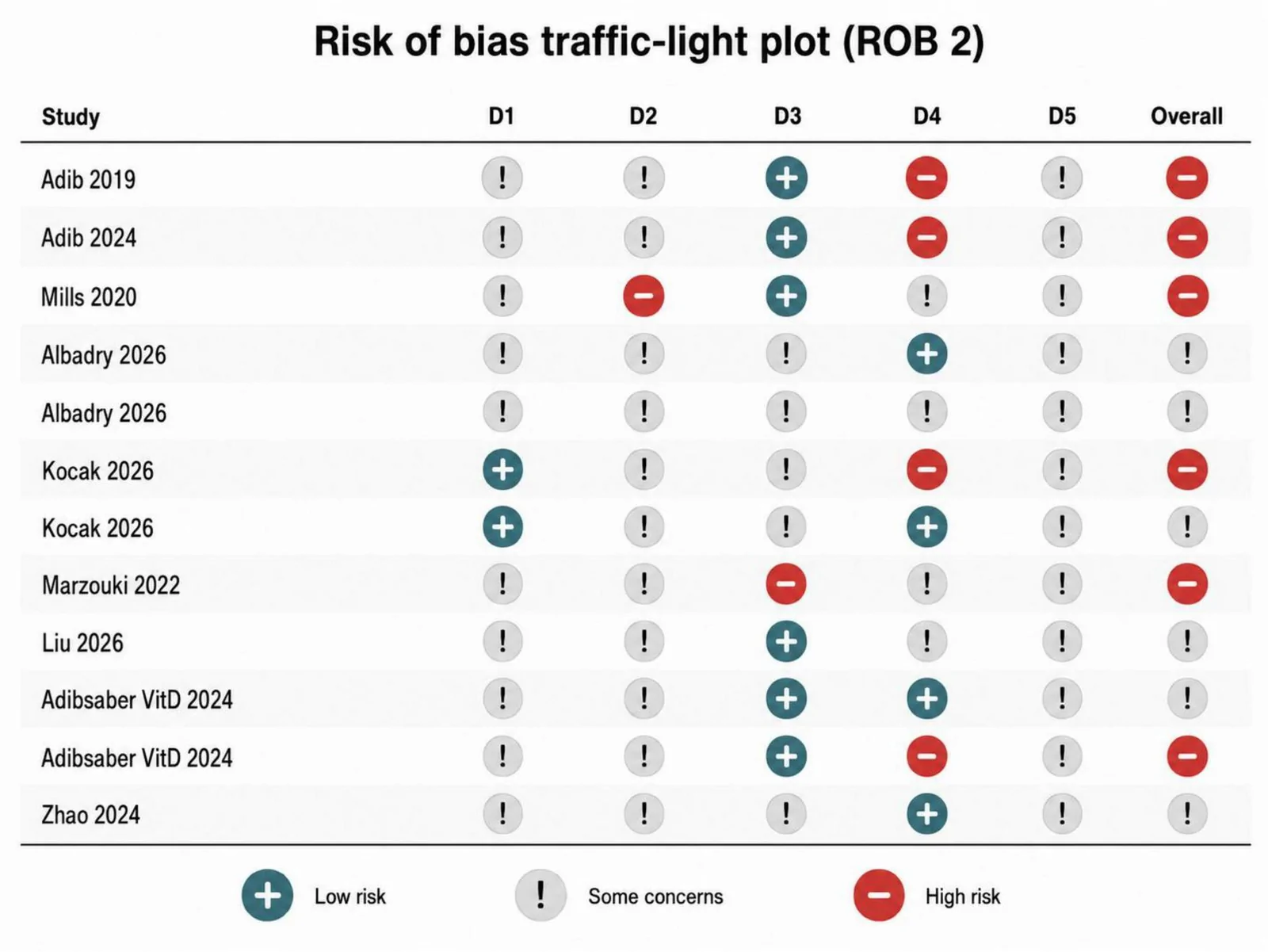
RoB 2 domain-level risk of bias assessment for included randomized controlled trials.

**FIGURE 3:**
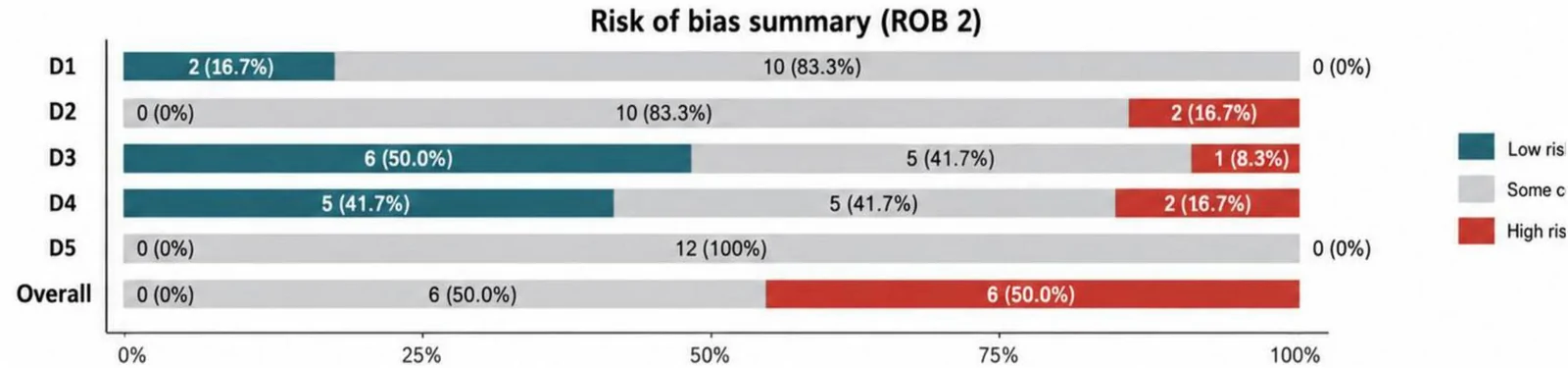
Overall risk of bias across studies for each RoB 2 domain.

The RoB 2 assessment results are shown in Figures 2, 3. Overall, the included studies had a relatively high risk of bias. Among the 12 outcome/report-level assessments, none were judged as low risk, while six were judged as having some concerns and six as high risk, each accounting for 50.0%. The main concerns were insufficient reporting of the randomization process, risk of deviations from intended interventions, difficulty in blinding outcome assessment for subjective outcomes, and uncertainty regarding selective reporting. The domain of missing outcome data was relatively better, although some studies still had attrition or insufficient handling of missing data. Therefore, the results should be interpreted with caution.

### 3.3 Overall ASD-related symptoms or behaviors

**FIGURE 4:**
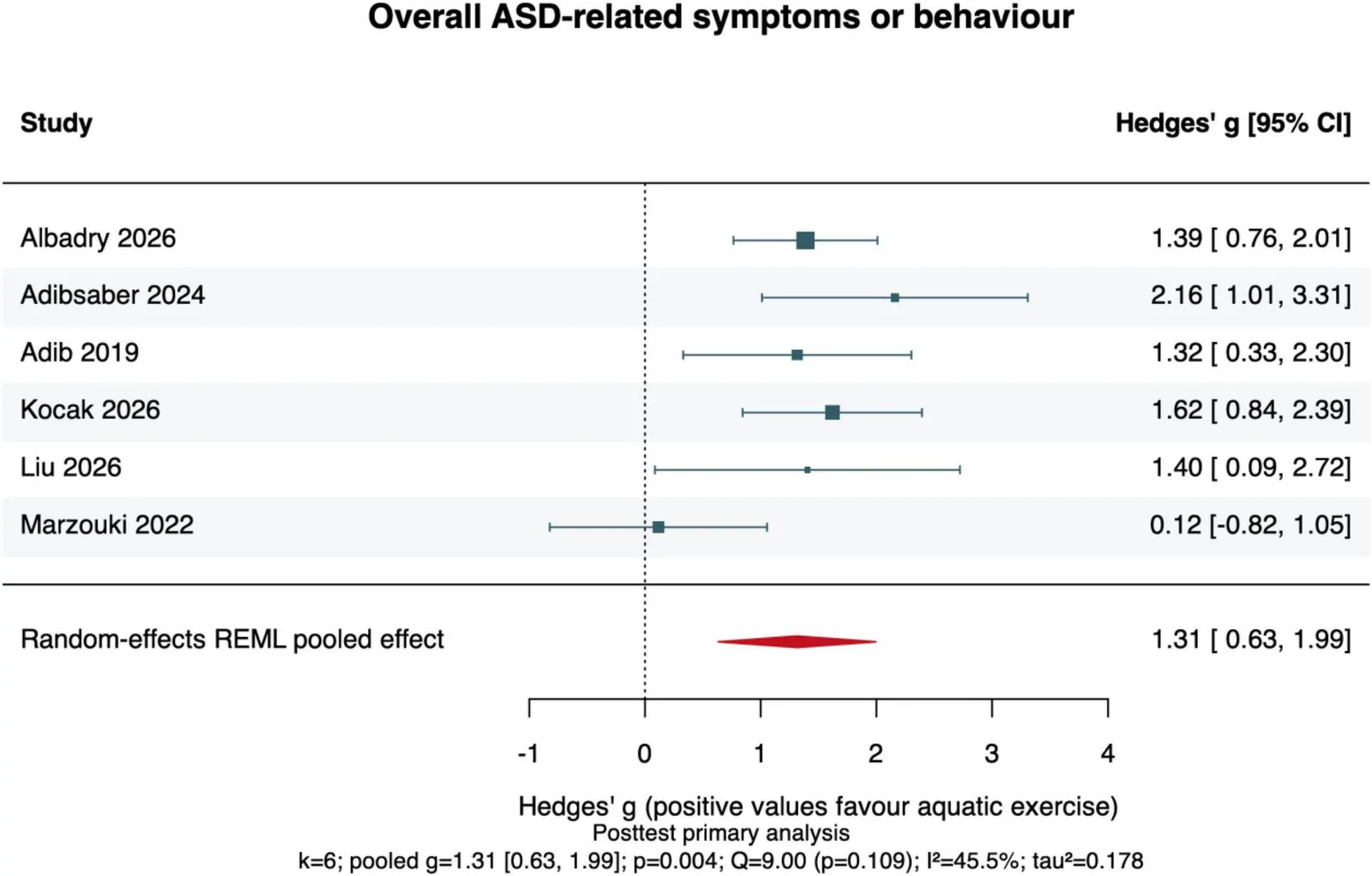
Forest plot of the effects of aquatic exercise on overall ASD-related symptoms or behaviors in children with ASD. Effect sizes are post-intervention Hedges’ g values, with positive values favoring aquatic exercise. The red diamond represents the pooled effect from the REML random-effects model.

A total of 6 independent studies involving 159 children were included for overall ASD-related symptoms or behaviors. The REML random-effects meta-analysis showed that post-intervention outcomes were better in the aquatic exercise group than in the control group (Figure 4 and Supplementary Table 4), with a pooled effect size of g = 1.31 (95% CI 0.63–1.99, *p* = 0.004). Between-study heterogeneity was moderate (*Q* = 9.00, *p* = 0.109; *I*^2^ = 45.5%; τ^2^ = 0.178).

After excluding each study one at a time, the pooled effects remained positive, ranging from 1.19 to 1.52, and none of the 95% CIs crossed the null line (Supplementary Table 6). The change-score analysis yielded *g* = 1.27 when *r* = 0.5 (95% CI 0.31–2.22, *p* = 0.019; *I*^2^ = 75.6%). When r was adjusted to 0.3 and 0.7, the pooled effects were 1.28 and 1.25, respectively, with no substantial change in the direction or statistical inference. However, heterogeneity in the change-score analysis was higher than that in the primary post-intervention analysis (Supplementary Table 5).

### 3.4 Broad motor or physical function

**FIGURE 5:**
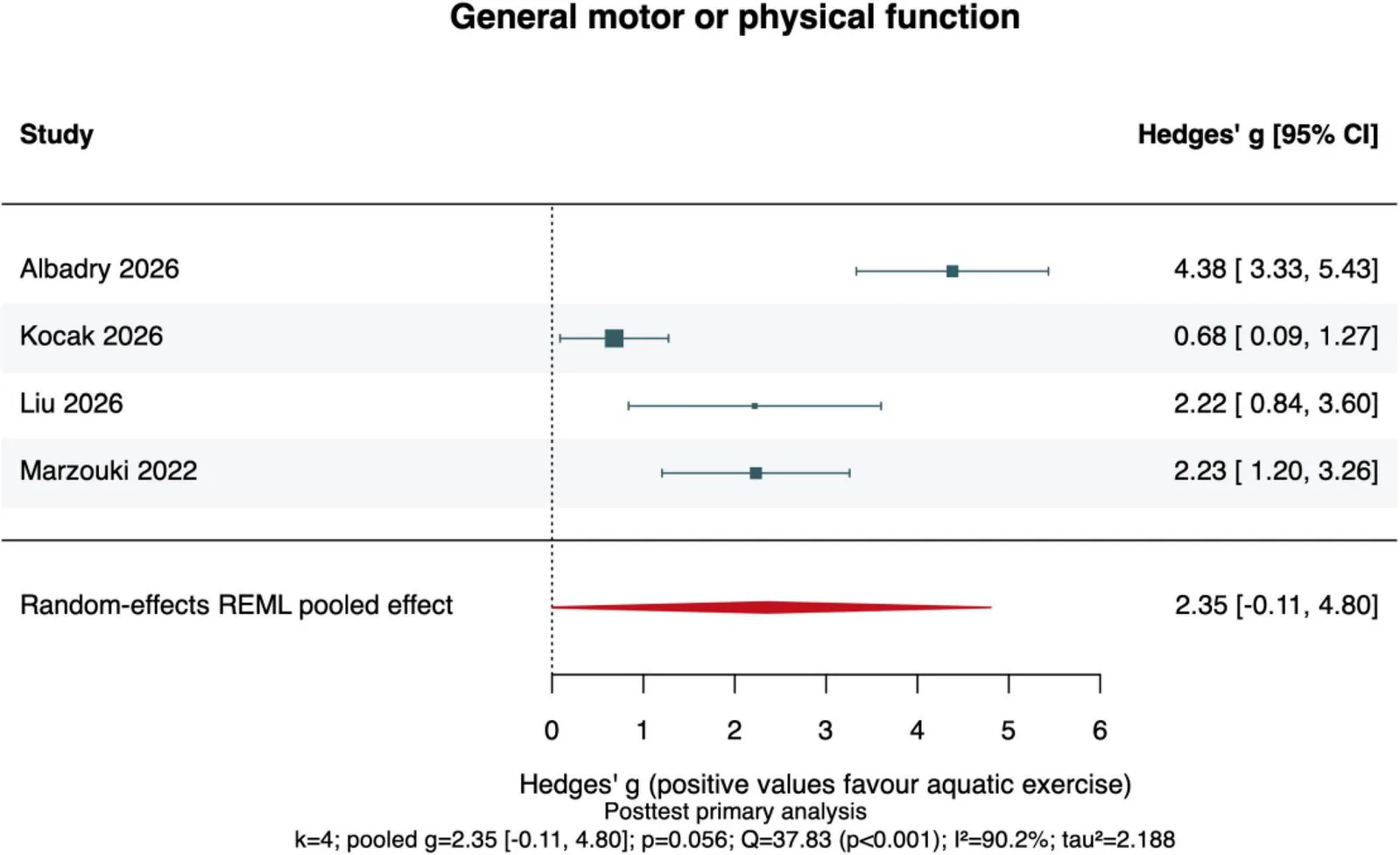
Forest plot of the effects of aquatic exercise on broad motor or physical function in children with ASD. Effect sizes are post-intervention Hedges’ g values, with positive values favoring aquatic exercise. The red diamond represents the pooled effect from the REML random-effects model.

A total of 4 independent studies involving 119 children were included for broad motor or physical function. The pooled effect from the random-effects model favored aquatic exercise (Figure 5; Supplementary Table 4), but the confidence interval crossed the null line (*g* = 2.35, 95% CI −0.11 to 4.80, *p* = 0.056). Between-study heterogeneity was very high (*Q* = 37.83, *p* < 0.001; *I*^2^ = 90.2%; τ^2^ = 2.188).

This result was sensitive to the assumed within-study outcome correlation coefficient. When ρ = 0.3, 0.5, and 0.7, the pooled effects were 2.48 (95% CI 0.01–4.94), 2.35 (95% CI −0.11 to 4.80), and 2.15 (95% CI −0.43 to 4.73), respectively (Supplementary Table 7). After excluding the Albadry study, in which the mean and standard deviation were estimated from the median and interquartile range, the pooled post-intervention effect decreased to *g* = 1.60 (95% CI −0.71 to 3.91, *p* = 0.096; *I*^2^ = 74.3%) (Supplementary Table 8). The change-score analysis yielded *g* = 3.36 when *r* = 0.5 (95% CI −2.33 to 9.05, *p* = 0.157; *I*^2^ = 97.9%) (Supplementary Table 5). Therefore, although the overall direction of the motor function results favored aquatic exercise, the effect size, statistical significance, and heterogeneity were unstable.

### 3.5 CARS/CARS-2 autism severity

**FIGURE 6:**
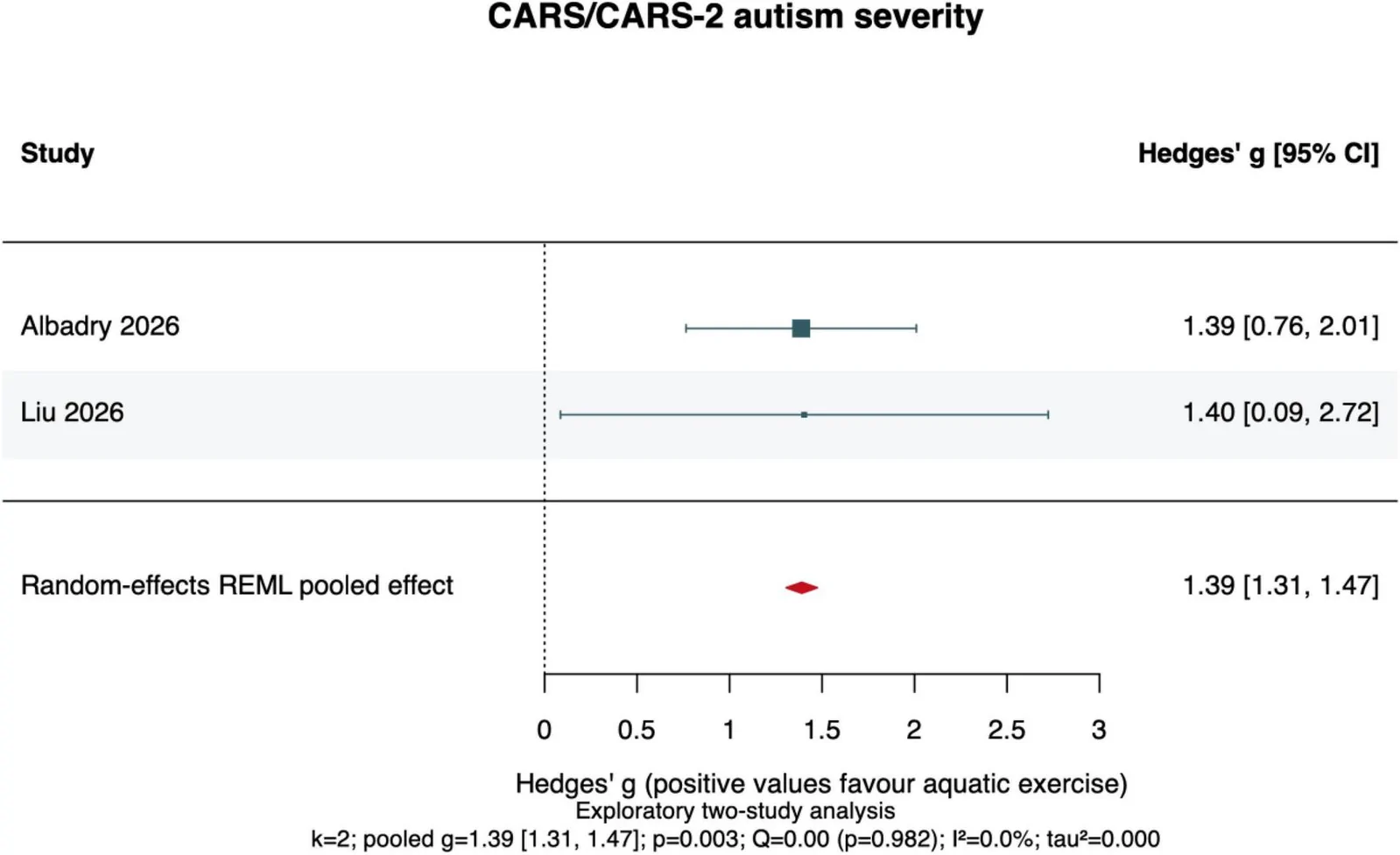
Exploratory forest plot of the effects of aquatic exercise on CARS/CARS-2 autism severity. Only two studies were included for this outcome, and the random-effects result should be interpreted together with the fixed-effect sensitivity analysis.

Only 2 studies involving 62 children were included for CARS/CARS-2 outcomes (Figure 6; Supplementary Table 4). The REML random-effects model yielded *g* = 1.39 (95% CI 1.31–1.47, *p* = 0.003; *I*^2^ = 0%), and the fixed-effect sensitivity analysis yielded *g* = 1.39 (95% CI 0.83–1.95, *p* < 0.001). The point estimates of the two studies were almost identical, resulting in an abnormally narrow Knapp–Hartung random-effects confidence interval without lower-bound correction. The results of the change-score analysis are presented in Supplementary Table 5. This finding should be interpreted as exploratory evidence, and the narrow interval should not be regarded as indicating high certainty.

### 3.6 Stereotyped or repetitive behaviors

**FIGURE 7:**
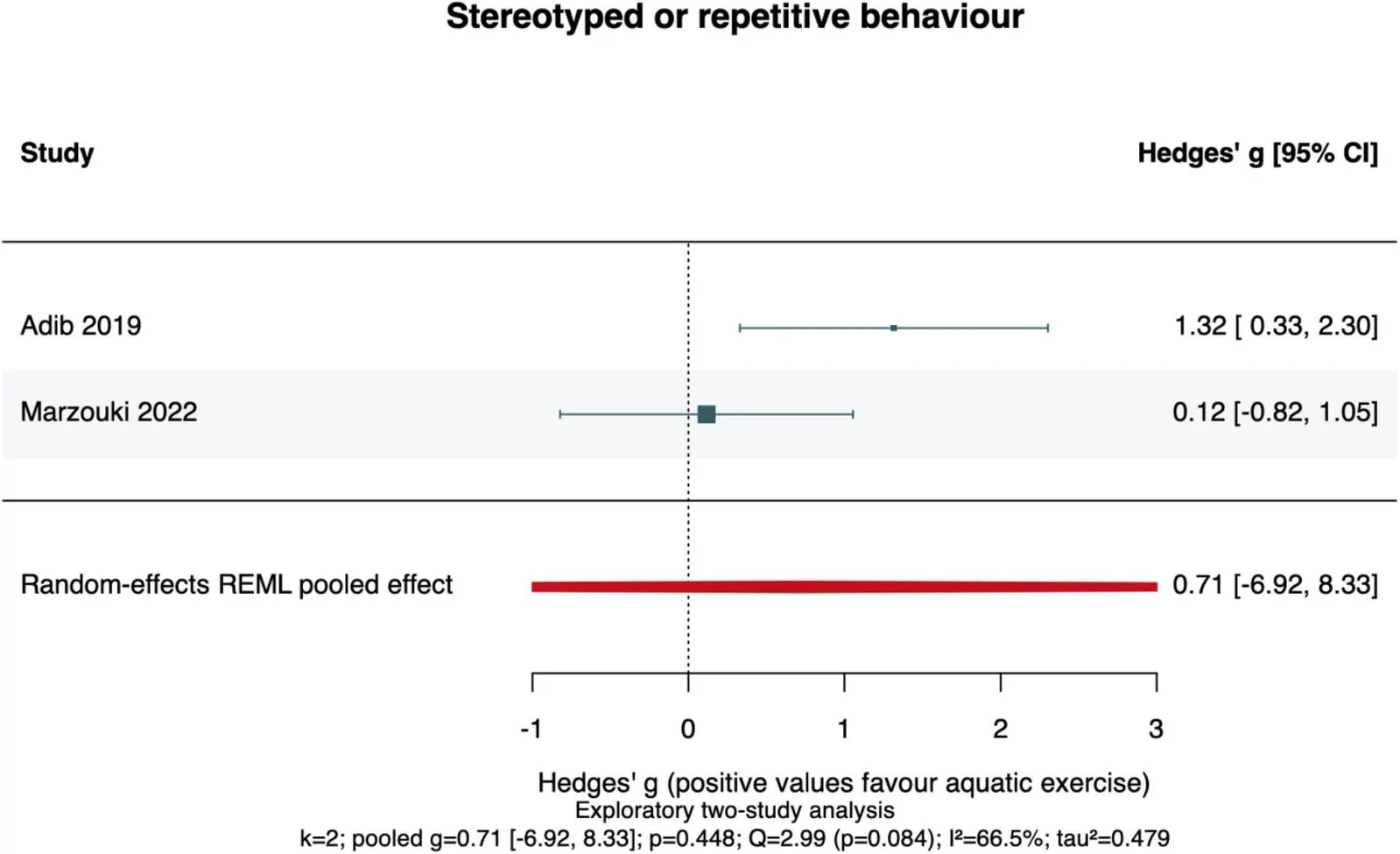
Exploratory forest plot of the effects of aquatic exercise on stereotyped or repetitive behaviors. Only two studies were included for this outcome, and the statistical inferences differed between the random-effects and fixed-effect models.

Only 2 studies involving 42 children were included for stereotyped or repetitive behaviors (Figure 7 and Supplementary Table 4). The random-effects result was *g* = 0.71 (95% CI −6.92 to 8.33, *p* = 0.448; *I*^2^ = 66.5%), whereas the fixed-effect sensitivity analysis yielded *g* = 0.69 (95% CI 0.01–1.37, *p* = 0.048). The results of the change-score analysis are presented in Supplementary Table 5. The statistical inferences differed between the two models, indicating that the current evidence is insufficient and unstable.

### 3.7 Social function or social impairment

**FIGURE 8:**
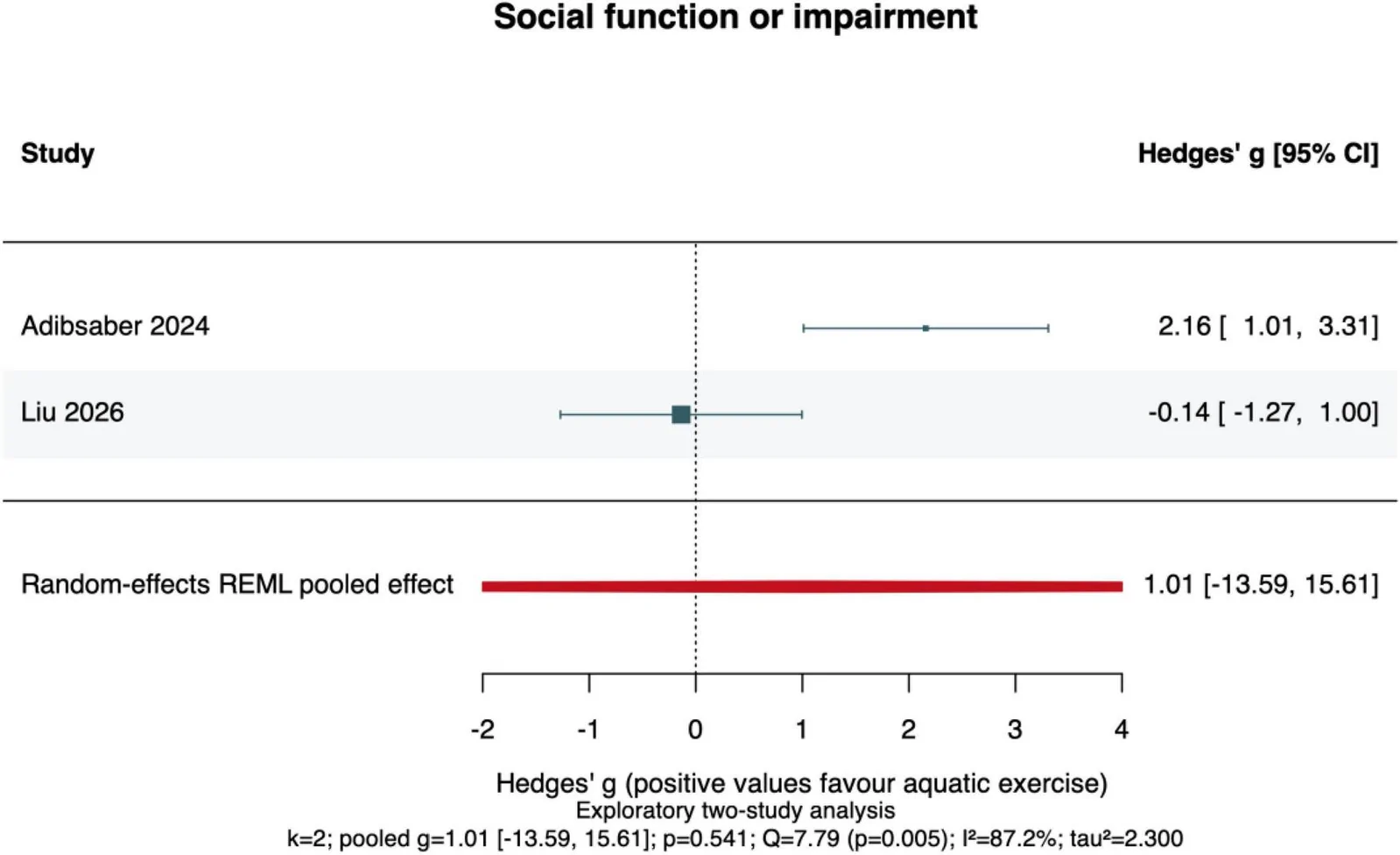
Exploratory forest plot of the effects of aquatic exercise on social function or social impairment. Only two studies were included for this outcome, with very high heterogeneity and inconsistent model-based inferences.

Only 2 studies involving 32 children were included for social function or social impairment (Figure 8 and Supplementary Table 4). The random-effects result was *g* = 1.01 (95% CI −13.59 to 15.61, *p* = 0.541; *I*^2^ = 87.2%), whereas the fixed-effect sensitivity analysis yielded g = 1.00 (95% CI 0.19–1.80, *p* = 0.016). The results of the change-score analysis are presented in Supplementary Table 5. Because the number of studies was very small, heterogeneity was very high, and model-based inferences were inconsistent, no definitive conclusion can be drawn.

### 3.8 Subgroup analysis

**FIGURE 9:**
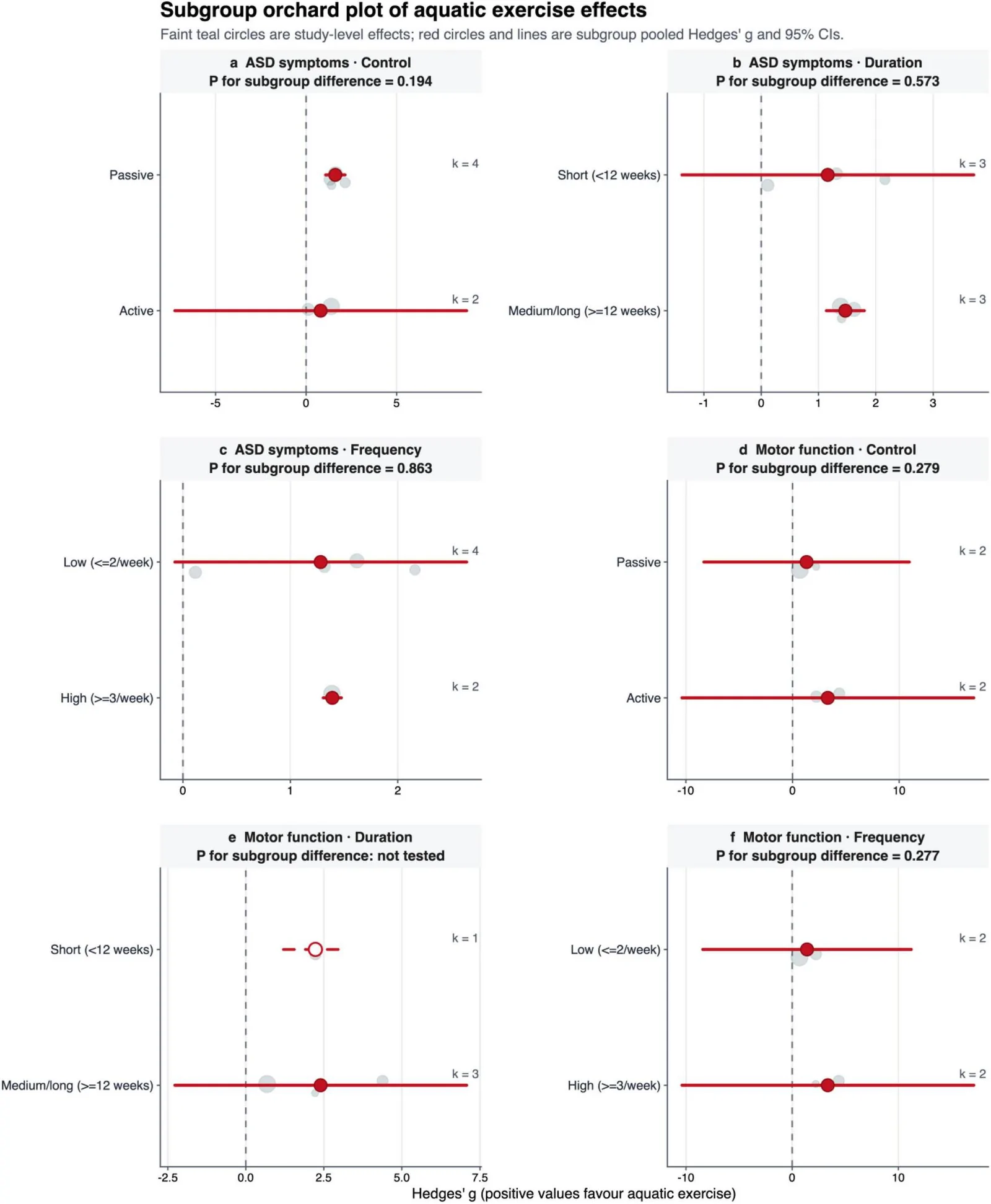
Combined subgroup orchard plot for overall ASD-related symptoms or behaviors and broad motor or physical function. Subgroup analyses are exploratory. Open circles and dashed intervals denote descriptive one-study subgroups. A significant within-subgroup estimate does not establish a subgroup difference; therefore, subgroup differences should be interpreted according to the displayed P for subgroup difference.

The subgroup analysis results for overall ASD-related symptoms or behaviors and broad motor or physical function are shown in Figure 9, and the formal tests for subgroup differences are presented in Supplementary Table 9. For overall ASD-related symptoms or behaviors, formal between-subgroup tests showed no statistically significant differences by control type, intervention duration, or training frequency, with P for subgroup difference values of 0.194, 0.573, and 0.863, respectively. For broad motor or physical function, between-subgroup differences by control type and training frequency were also not statistically significant, with P values of 0.279 and 0.277, respectively. In the duration subgroup analysis for motor function, the short-term subgroup included only 1 study; therefore, no formal between-subgroup test was conducted.

Although some within-subgroup pooled effects reached statistical significance, none of the formal subgroup difference tests identified a reliable moderating effect. Given the limited number of studies and low statistical power, these analyses should be regarded as exploratory.

### 3.9 Other outcomes and small-study effects

Sleep, mental health, emotion regulation, sensory integration, aquatic skills, quality of life, executive function, IL-6, IL-10, and BDNF were each reported by only 1 independent study. Therefore, conventional meta-analyses were not performed for these outcomes, and they were summarized narratively only (Supplementary Table 10). The mental health-related behavioral outcomes in Mills 2020 were summarized narratively because paired information for the crossover design was insufficient. The executive function and BDNF outcomes in Zhao et al. (2024) were not extracted as precise post-intervention means and standard deviations from bar charts.

**FIGURE 10:**
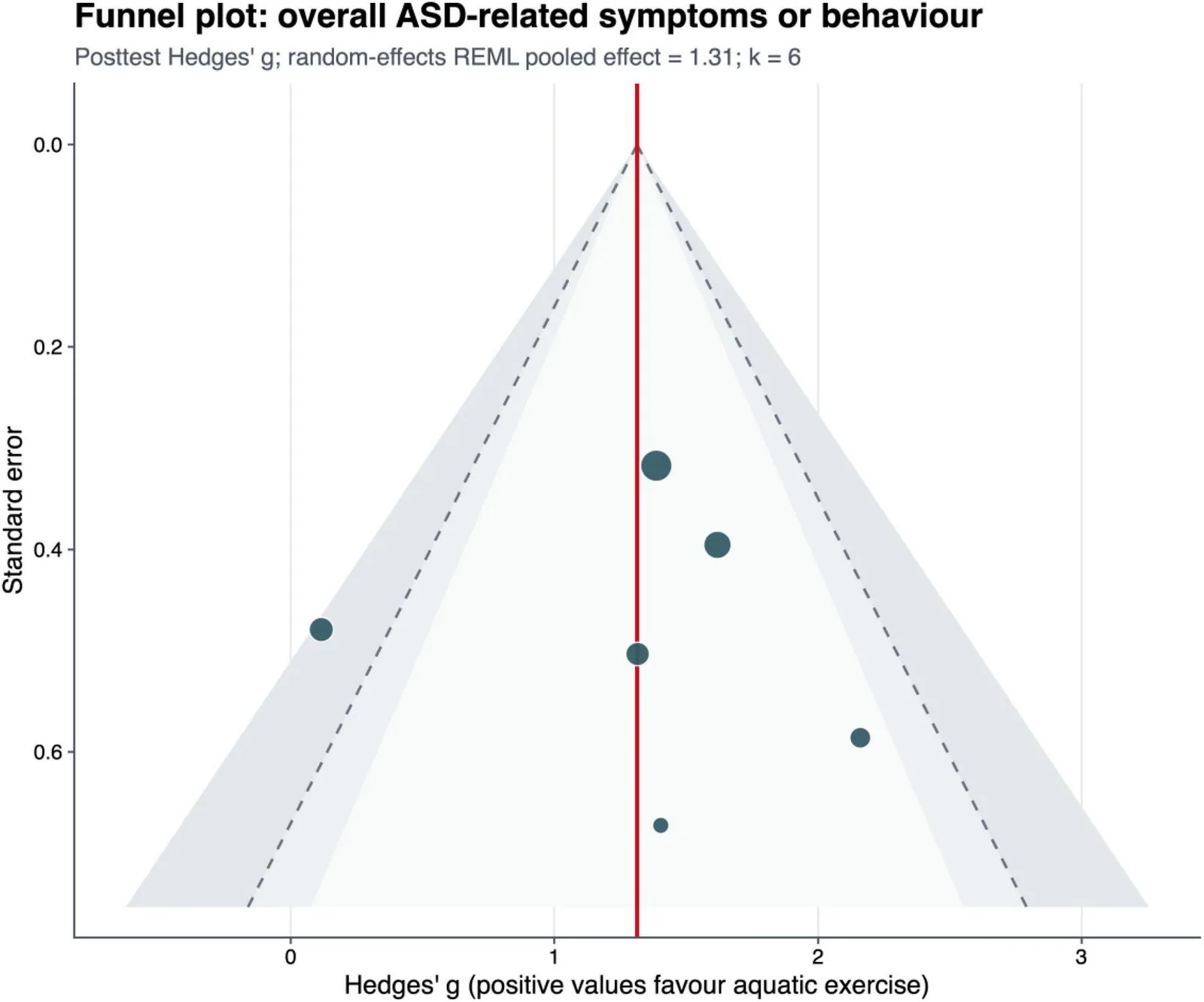
Contour-enhanced funnel plot for overall ASD-related symptoms or behaviors. This plot included only six studies and was used for descriptive purposes only. Egger’s test was not performed, and no judgment regarding the presence or absence of publication bias was made based on this plot.

A contour-enhanced funnel plot was generated for overall ASD-related symptoms or behaviors (Figure 10). Because the plot included only 6 studies, it was used only to describe the distribution of effect sizes and standard errors. Egger’s test was not performed, and the funnel plot was not used to judge the presence or absence of publication bias. Nevertheless, unpublished studies with null or unfavorable findings may exist, and the pooled effect may therefore be overestimated.

## 4 Discussion

### 4.1 Main findings

This systematic review and meta-analysis showed that aquatic exercise had a favorable effect on overall ASD-related symptoms or behaviors in children with ASD. The pooled effect remained positive after leave-one-out analyses, and the change-score sensitivity analyses showed a consistent direction, suggesting a relatively stable improvement signal. In contrast, broad motor or physical function also showed a favorable direction of effect, but the confidence interval crossed the null line, heterogeneity was high, and the magnitude and precision of the effect varied across sensitivity analyses, indicating considerable uncertainty. CARS/CARS-2, stereotyped behavior, and social functioning were each based on only 2 studies, and the findings were unstable across analytical models and susceptible to the influence of individual studies. These findings should therefore be interpreted as exploratory. Subgroup analyses did not identify reliable moderating effects of control type, intervention duration, or training frequency.

### 4.2 Relationship with existing evidence and potential explanations

The favorable findings for overall ASD-related symptoms or behaviors in this study are generally consistent with the broader literature on exercise interventions. Previous meta-analyses and systematic reviews have reported varying degrees of potential benefits for behavioral outcomes, social communication, executive function, sleep, and motor function (Healy et al., 2018; Ruggeri et al., 2019; Huang et al., 2020; Monteiro et al., 2022; Ji et al., 2023; Kou et al., 2024; Wu et al., 2024; Wang et al., 2025). Accordingly, the present findings support aquatic exercise as a potentially beneficial adjunctive intervention, but do not establish a definitive therapeutic effect across all ASD-related domains. The generally favorable direction observed for broad motor outcomes is also consistent with the common presence of motor coordination and gross motor difficulties in children with ASD (Fournier et al., 2010; Wang et al., 2022; Kangarani-Farahani et al., 2023; Miller et al., 2023). However, the considerable heterogeneity indicates that benefits may differ across motor constructs, intervention formats, and participant characteristics.

Previous reviews of aquatic exercise have generally suggested that such programs are feasible and may improve motor ability, aquatic skills, or some social behavioral outcomes (Kumar et al., 2014; Naumann et al., 2021; Rohn et al., 2021; Ogonowska-Slodownik et al., 2024; van t Hooft et al., 2024). Direct trials have also covered a wide range of outcomes, including gross motor function, balance, behavior, social functioning, aquatic skills, quality of life, executive function, and biomarkers (Ansari et al., 2020; Mills et al., 2020; Güeita-Rodríguez et al., 2021; Marzouki et al., 2022; Vodakova et al., 2022; Zhao et al., 2024; Albadry et al., 2026; Koçak et al., 2026). However, earlier evidence often came from uncontrolled or small-sample studies, and recent randomized controlled trials still have several limitations, including small sample sizes, inconsistent control conditions, completer analyses, and substantial differences in outcome instruments. The present synthesis therefore strengthens the evidence for a favorable effect on overall ASD-related symptoms or behaviors, while also showing that this finding cannot be generalized to all motor, behavioral, or social outcomes.

The aquatic environment may modify mechanical loading, postural control, and sensory input through buoyancy, hydrostatic pressure, water resistance, and temperature (Becker, 2009). Buoyancy reduces effective weight-bearing, while hydrostatic pressure and water resistance may support proprioceptive input, postural stability, and controlled movement practice. At the same time, changes in body position, water flow, and balance demands may provide concurrent proprioceptive, tactile, and vestibular stimulation. The integration of these sensory inputs may support body awareness, postural control, and motor planning, which are frequently challenging for children with ASD. Repeated practice in a relatively supportive and predictable environment may also facilitate the organization and execution of purposeful movements. However, these effects depend on factors such as water depth, immersion level, body position, movement velocity, and water temperature, which were insufficiently reported in most included studies. Therefore, these aquatic factors could not be examined as potential moderators.

Structured aquatic exercise programs may also influence behavioral and social outcomes through arousal regulation and motor–social coupling. Predictable routines, visual cues, instructor feedback, and repeated movement sequences may reduce task uncertainty and help children maintain an appropriate level of arousal and attention. Exercise-related regulation of executive function, sleep, and inflammation-related processes may provide additional pathways (Liang et al., 2021, 2024; Toscano et al., 2021; Su et al., 2022). Furthermore, aquatic activities often require imitation, turn-taking, shared attention, and coordinated movement with instructors or peers. Improvements in motor planning and movement confidence may therefore increase opportunities for social engagement and participation. This potential motor–social coupling is consistent with the reported association between motor ability and social skills in children with ASD (Wang et al., 2022), and improvements in motor ability may theoretically increase opportunities for children with ASD to participate in peer play and daily activities. However, the pooled analysis of social functioning in the present study included only 2 studies and showed high heterogeneity. Therefore, it cannot be inferred that improvements in motor function necessarily translate into social benefits. Similarly, the BDNF changes reported by Zhao et al. (2024) may provide clues for future mechanistic research, but a single study is insufficient to establish a causal chain linking aquatic exercise, BDNF changes, and clinical improvement. These mechanisms should therefore be regarded as integrated and biologically plausible explanations rather than causal pathways confirmed by the current evidence.

### 4.3 Instability of the results and moderator analyses

The uncertainty in the motor function results may mainly arise from differences in outcome constructs and measurement tools. In this study, motor function outcomes combined different indicators, including the PDMS-2 gross motor quotient, TGMD-2 gross motor skills, balance, flexibility, and grip strength. These outcomes represent distinct neurodevelopmental and physical-function constructs and should not be expected to respond uniformly to aquatic exercise. Gross motor skills mainly reflect motor development and movement competence, balance reflects postural control, flexibility reflects range of motion, and grip strength reflects muscular strength. Differences in participant age, assessment methods, control conditions, and training content may have further contributed to the high heterogeneity. Broader reviews of exercise interventions have generally reported favorable directions, but they have also observed substantial heterogeneity across intervention protocols and outcome measures (Healy et al., 2018; Ruggeri et al., 2019; Monteiro et al., 2022; Ji et al., 2023; Wang et al., 2025). Therefore, the pooled estimate should be interpreted only as a broad summary of motor and physical function rather than as evidence of a consistent effect across all motor domains. In the present study, the statistical inference varied under different within-study correlation assumptions, and excluding the study with estimated data reduced the pooled effect. Taken together, these findings indicate a favorable but uncertain signal rather than conclusive evidence that aquatic exercise improves motor function.

For outcomes based on only 2 studies, such as CARS/CARS-2, stereotyped behavior, and social function, the results are more susceptible to model specification and the influence of individual studies. The unusually narrow confidence interval for CARS/CARS-2 should not be interpreted as indicating high certainty, because estimates based on very few studies may be artificially precise when between-study heterogeneity is estimated as close to zero (Röver et al., 2015). Similarly, the inconsistent conclusions from the fixed-effect and random-effects models for stereotyped behavior and social function further indicate that the evidence remains insufficient. Favorable signals for stereotyped behavior and social communication have been reported in the broader exercise literature (Bremer et al., 2016; Jia et al., 2023; Kou et al., 2024; Wang et al., 2025), but such indirect evidence cannot replace robust aquatic-exercise-specific evidence.

Subgroup and dose-related findings also require cautious interpretation. This study did not find formal between-subgroup differences according to control type, intervention duration, or training frequency. It should be emphasized that statistical significance within a subgroup does not indicate a statistically significant difference between subgroups. Moreover, each subgroup included only 1–4 studies, and intervention formats, outcome measures, and dose reporting varied considerably. Incomplete reporting of training frequency, total sessions, and intervention dose may also have reduced the accuracy of subgroup classification. Therefore, the current data cannot be used to determine the optimal dose of aquatic exercise, identify reliable moderators, or establish that a specific control condition or intervention arrangement is more effective.

### 4.4 Clinical implications

The pooled effect of *g* = 1.31 indicates a large standardized group-level difference in favor of aquatic exercise. However, because different symptom and behavioral scales were combined, this effect cannot be directly translated into a specific clinical score change or interpreted as clinically meaningful improvement for every child. Aquatic exercise should therefore be considered an adjunct to, rather than a replacement for, behavioral, educational, occupational, physical, or communication-based interventions. It may be incorporated into individualized rehabilitation plans targeting behavioral regulation, motor function, physical activity participation, or water competence. The current evidence does not identify which children benefit most; however, aquatic exercise may be considered for children who can safely tolerate the aquatic environment and have relevant rehabilitation goals. Before participation, children with ASD should undergo an appropriate health and safety assessment to identify potential medical contraindications and determine the level of supervision required during aquatic exercise. Intervention adherence and feasibility may depend on children’s sensory tolerance and water competence, caregiver support, access to aquatic facilities, and the availability of trained personnel. Attendance, treatment response, adverse events, and reasons for withdrawal should therefore be monitored and reported.

### 4.5 Strengths and limitations

A strength of this study is that it attempted to minimize confusion in effect direction and repeated weighting. We prespecified a unified effect direction and used post-test values as the primary analysis, avoiding the mixture of post-test standardized mean differences and change-score effects within the same model. We also removed duplicate trial families and addressed multi-arm trials and multiple correlated outcomes within the same study. In addition, targeted sensitivity analyses were conducted for estimated data, within-study correlation coefficients, and outcomes based on only 2 studies, thereby improving the interpretability of the pooled results.

This study also has several limitations. First, the number of included studies and sample sizes were limited, and most outcomes included only a small number of studies. As a result, heterogeneity estimates, overall effects, and subgroup results may all be unstable. Second, there were substantial differences in aquatic exercise protocols, control conditions, and outcome tools. In particular, motor function outcomes included different constructs such as gross motor skills, balance, flexibility, and grip strength, which limits the interpretation of the pooled results. Moreover, most studies did not adequately report water depth, immersion level, water temperature, or other aquatic parameters, preventing further analysis of hydrostatic pressure and buoyancy-related unloading as potential moderators. Third, some data were derived from estimation, and change-score analyses also depended on assumptions about unreported pre–post correlations; therefore, these analyses cannot replace true paired data. Finally, some studies had methodological limitations related to randomization, allocation concealment, outcome measurement, handling of attrition, and selective reporting. Meanwhile, insufficient data on long-term follow-up, safety, and adherence limited the assessment of sustained effects and practical implementation value.

Future studies should conduct adequately powered, preregistered, multicenter randomized controlled trials and should clearly report random sequence generation, allocation concealment, blinding of outcome assessors, adherence, and adverse events. Aquatic exercise parameters, including water depth, immersion level, water temperature, and exercise intensity, should also be reported in a standardized manner. Outcome design should distinguish overall symptoms or behaviors, specific autism-related scales, gross motor skills, balance, strength, social function, sleep, and quality of life. Baseline values, post-test values, change scores, and pre–post correlation information should also be reported as completely as possible. Only after more independent studies have accumulated will it be appropriate to further examine potential moderators such as intervention duration, frequency, session length, control type, and child characteristics.

## 5 Conclusion

Current randomized controlled evidence suggests that aquatic exercise may improve overall ASD-related symptoms or behaviors in children with ASD, and this result was relatively stable in leave-one-out analyses and change-score analyses using different pre–post correlation coefficients. Broad motor or physical function showed a favorable direction, but the result remains inconclusive because of high heterogeneity, assumptions about within-study correlation coefficients, and the influence of estimated data. Outcomes such as CARS/CARS-2, stereotyped behavior, and social function were each based on only a small number of studies and should be regarded as exploratory evidence. The current evidence supports aquatic exercise as a potential adjunctive physical activity option within comprehensive support for children with ASD, but it remains insufficient to determine the optimal intervention dose, reliable moderators, or long-term benefits.

## Data Availability

The original contributions presented in the study are included in the article/Supplementary material, further inquiries can be directed to the corresponding author.
