# Supplementary material for: Effects of aquatic exercise on symptoms, behaviors, and motor function in children with autism spectrum disorder: a systematic review and meta-analysis
Source: Front Behav Neurosci. 2026 Aug 12;20:1927011. doi: 10.3389/fnbeh.2026.1927011 (PMC13506929; doi:10.3389/fnbeh.2026.1927011)
Supplement: Supplementary file 1 [file Data_Sheet_1.docx]

**SUPPLEMENTAL MATERIAL**

Table S1 PRISMA checklist of items to include when reporting a systematic review

| **Section and Topic** | **Item #** | **Checklist item** | **Location where item is reported** |
| --- | --- | --- | --- |
| **TITLE** | | |  |
| Title | 1 | Identify the report as a systematic review. | p.1 |
| **ABSTRACT** | | |  |
| Abstract | 2 | See the PRISMA 2020 for Abstracts checklist. | p.2 |
| **INTRODUCTION** | | |  |
| Rationale | 3 | Describe the rationale for the review in the context of existing knowledge. | p.3 |
| Objectives | 4 | Provide an explicit statement of the objective(s) or question(s) the review addresses. | p.3 |
| **METHODS** | | |  |
| Eligibility criteria | 5 | Specify the inclusion and exclusion criteria for the review and how studies were grouped for the syntheses. | p.5 |
| Information sources | 6 | Specify all databases, registers, websites, organisations, reference lists and other sources searched or consulted to identify studies. Specify the date when each source was last searched or consulted. | p.4 |
| Search strategy | 7 | Present the full search strategies for all databases, registers and websites, including any filters and limits used. | p.4 |
| Selection process | 8 | Specify the methods used to decide whether a study met the inclusion criteria of the review, including how many reviewers screened each record and each report retrieved, whether they worked independently, and if applicable, details of automation tools used in the process. | p.4-5 |
| Data collection process | 9 | Specify the methods used to collect data from reports, including how many reviewers collected data from each report, whether they worked independently, any processes for obtaining or confirming data from study investigators, and if applicable, details of automation tools used in the process. | p.5-6 |
| Data items | 10a | List and define all outcomes for which data were sought. Specify whether all results that were compatible with each outcome domain in each study were sought (e.g. for all measures, time points, analyses), and if not, the methods used to decide which results to collect. | p.5 |
|  | 10b | List and define all other variables for which data were sought (e.g. participant and intervention characteristics, funding sources). Describe any assumptions made about any missing or unclear information. | p.5 |
| Study risk of bias assessment | 11 | Specify the methods used to assess risk of bias in the included studies, including details of the tool(s) used, how many reviewers assessed each study and whether they worked independently, and if applicable, details of automation tools used in the process. | p.6 |
| Effect measures | 12 | Specify for each outcome the effect measure(s) (e.g. risk ratio, mean difference) used in the synthesis or presentation of results. | p.6 |
| Synthesis methods | 13a | Describe the processes used to decide which studies were eligible for each synthesis (e.g. tabulating the study intervention characteristics and comparing against the planned groups for each synthesis (item #5)). | p.6 |
|  | 13b | Describe any methods required to prepare the data for presentation or synthesis, such as handling of missing summary statistics, or data conversions. | p.6 |
|  | 13c | Describe any methods used to tabulate or visually display results of individual studies and syntheses. | p.6 |
|  | 13d | Describe any methods used to synthesize results and provide a rationale for the choice(s). If meta-analysis was performed, describe the model(s), method(s) to identify the presence and extent of statistical heterogeneity, and software package(s) used. | p.6-7 |
|  | 13e | Describe any methods used to explore possible causes of heterogeneity among study results (e.g. subgroup analysis, meta-regression). | p.7 |
|  | 13f | Describe any sensitivity analyses conducted to assess robustness of the synthesized results. | p.8 |
| Reporting bias assessment | 14 | Describe any methods used to assess risk of bias due to missing results in a synthesis (arising from reporting biases). | p.6 |
| Certainty assessment | 15 | Describe any methods used to assess certainty (or confidence) in the body of evidence for an outcome. | p.8;  Figure 2 |
| **RESULTS** | | |  |
| Study selection | 16a | Describe the results of the search and selection process, from the number of records identified in the search to the number of studies included in the review, ideally using a flow diagram. | p.9;  Figure 1 |
|  | 16b | Cite studies that might appear to meet the inclusion criteria, but which were excluded, and explain why they were excluded. | p.9 |
| Study characteristics | 17 | Cite each included study and present its characteristics. | Table S7 |
| Risk of bias in studies | 18 | Present assessments of risk of bias for each included study. | Figure S8-9 |
| Results of individual studies | 19 | For all outcomes, present, for each study: (a) summary statistics for each group (where appropriate) and (b) an effect estimate and its precision (e.g. confidence/credible interval), ideally using structured tables or plots. | Figure 2  Figure S1-7 |
| Results of syntheses | 20a | For each synthesis, briefly summarise the characteristics and risk of bias among contributing studies. | p.9 |
|  | 20b | Present results of all statistical syntheses conducted. If meta-analysis was done, present for each the summary estimate and its precision (e.g. confidence/credible interval) and measures of statistical heterogeneity. If comparing groups, describe the direction of the effect. | p.9;  Figure 2 |
|  | 20c | Present results of all investigations of possible causes of heterogeneity among study results. | p.10-12 |
|  | 20d | Present results of all sensitivity analyses conducted to assess the robustness of the synthesized results. | p.13 |
| Reporting biases | 21 | Present assessments of risk of bias due to missing results (arising from reporting biases) for each synthesis assessed. | p.12 |
| Certainty of evidence | 22 | Present assessments of certainty (or confidence) in the body of evidence for each outcome assessed. | p.10 |
| **DISCUSSION** | | |  |
| Discussion | 23a | Provide a general interpretation of the results in the context of other evidence. | p.14-23 |
|  | 23b | Discuss any limitations of the evidence included in the review. | p.22 |
|  | 23c | Discuss any limitations of the review processes used. | p.22 |
|  | 23d | Discuss implications of the results for practice, policy, and future research. | p.22-23 |
| **OTHER INFORMATION** | | |  |
| Registration and protocol | 24a | Provide registration information for the review, including register name and registration number, or state that the review was not registered. | p.4 |
|  | 24b | Indicate where the review protocol can be accessed, or state that a protocol was not prepared. | p.4 |
|  | 24c | Describe and explain any amendments to information provided at registration or in the protocol. | p.4 |
| Support | 25 | Describe sources of financial or non-financial support for the review, and the role of the funders or sponsors in the review. | p.24 |
| Competing interests | 26 | Declare any competing interests of review authors. | p.24 |
| Availability of data, code and other materials | 27 | Report which of the following are publicly available and where they can be found: template data collection forms; data extracted from included studies; data used for all analyses; analytic code; any other materials used in the review. | p.24 |

Table S2. Complete Search Strategies for Each Database

| **Database** | **Search Strategy** |
| --- | --- |
| PubMed | ("Autism Spectrum Disorder"[Mesh] OR "Autistic Disorder"[Mesh] OR "autism spectrum disorder"[tiab] OR "autism spectrum disorders"[tiab] OR ASD[tiab] OR autism[tiab] OR autistic[tiab] OR "autistic disorder"[tiab] OR "autistic disorders"[tiab] OR Asperger*[tiab] OR "pervasive developmental disorder"[tiab] OR "pervasive developmental disorders"[tiab] OR PDD[tiab] OR "PDD-NOS"[tiab] OR "childhood autism"[tiab]) AND ("Hydrotherapy"[Mesh] OR "Swimming"[Mesh] OR hydrotherap*[tiab] OR aquatic[tiab] OR "aquatic therapy"[tiab] OR "aquatic therapies"[tiab] OR "aquatic exercise"[tiab] OR "aquatic exercises"[tiab] OR "aquatic intervention"[tiab] OR "aquatic interventions"[tiab] OR "aquatic program"[tiab] OR "aquatic programs"[tiab] OR "aquatic programme"[tiab] OR "aquatic programmes"[tiab] OR "aquatic training"[tiab] OR "water-based"[tiab] OR "water based"[tiab] OR "water exercise"[tiab] OR "water exercises"[tiab] OR "water training"[tiab] OR "pool-based"[tiab] OR "pool based"[tiab] OR "pool therapy"[tiab] OR "pool exercise"[tiab] OR "pool exercises"[tiab] OR swimming[tiab] OR "swim training"[tiab] OR "swimming program"[tiab] OR "swimming programs"[tiab] OR "swimming programme"[tiab] OR "swimming programmes"[tiab] OR Halliwick[tiab] OR "multisystem aquatic therapy"[tiab]) AND (randomized controlled trial[pt] OR controlled clinical trial[pt] OR clinical trial[pt] OR random*[tiab] OR randomly[tiab] OR trial[tiab] OR controlled[tiab] OR "control group"[tiab] OR "control groups"[tiab] OR assign*[tiab] OR allocat*[tiab]) |
| Embase | ('autism spectrum disorder'/exp OR 'autistic disorder'/exp OR 'asperger syndrome'/exp OR 'pervasive developmental disorder'/exp OR 'autism spectrum disorder':ti,ab OR 'autism spectrum disorders':ti,ab OR ASD:ti,ab OR autism:ti,ab OR autistic:ti,ab OR 'autistic disorder':ti,ab OR 'autistic disorders':ti,ab OR Asperger*:ti,ab OR 'pervasive developmental disorder':ti,ab OR 'pervasive developmental disorders':ti,ab OR PDD:ti,ab OR 'PDD-NOS':ti,ab OR 'childhood autism':ti,ab) AND ('hydrotherapy'/exp OR 'swimming'/exp OR hydrotherap*:ti,ab OR aquatic:ti,ab OR 'aquatic therapy':ti,ab OR 'aquatic therapies':ti,ab OR 'aquatic exercise':ti,ab OR 'aquatic exercises':ti,ab OR 'aquatic intervention':ti,ab OR 'aquatic interventions':ti,ab OR 'aquatic program':ti,ab OR 'aquatic programs':ti,ab OR 'aquatic programme':ti,ab OR 'aquatic programmes':ti,ab OR 'aquatic training':ti,ab OR 'water-based':ti,ab OR 'water based':ti,ab OR 'water exercise':ti,ab OR 'water exercises':ti,ab OR 'water training':ti,ab OR 'pool-based':ti,ab OR 'pool based':ti,ab OR 'pool therapy':ti,ab OR 'pool exercise':ti,ab OR 'pool exercises':ti,ab OR swimming:ti,ab OR 'swim training':ti,ab OR 'swimming program':ti,ab OR 'swimming programs':ti,ab OR 'swimming programme':ti,ab OR 'swimming programmes':ti,ab OR Halliwick:ti,ab OR 'multisystem aquatic therapy':ti,ab) AND ('randomized controlled trial'/exp OR 'controlled clinical trial'/exp OR 'clinical trial'/exp OR random*:ti,ab OR randomly:ti,ab OR trial:ti,ab OR controlled:ti,ab OR 'control group':ti,ab OR 'control groups':ti,ab OR assign*:ti,ab OR allocat*:ti,ab) AND ([embase]/lim |
| Cochrane Library | ([mh "Autism Spectrum Disorder"] OR [mh "Autistic Disorder"] OR "autism spectrum disorder":ti,ab,kw OR "autism spectrum disorders":ti,ab,kw OR ASD:ti,ab,kw OR autism:ti,ab,kw OR autistic:ti,ab,kw OR "autistic disorder":ti,ab,kw OR "autistic disorders":ti,ab,kw OR Asperger*:ti,ab,kw OR "pervasive developmental disorder":ti,ab,kw OR "pervasive developmental disorders":ti,ab,kw OR PDD:ti,ab,kw OR "PDD-NOS":ti,ab,kw OR "childhood autism":ti,ab,kw) AND ([mh Hydrotherapy] OR [mh Swimming] OR hydrotherap*:ti,ab,kw OR aquatic:ti,ab,kw OR "aquatic therapy":ti,ab,kw OR "aquatic therapies":ti,ab,kw OR "aquatic exercise":ti,ab,kw OR "aquatic exercises":ti,ab,kw OR "aquatic intervention":ti,ab,kw OR "aquatic interventions":ti,ab,kw OR "aquatic program":ti,ab,kw OR "aquatic programs":ti,ab,kw OR "aquatic programme":ti,ab,kw OR "aquatic programmes":ti,ab,kw OR "aquatic training":ti,ab,kw OR "water-based":ti,ab,kw OR "water based":ti,ab,kw OR "water exercise":ti,ab,kw OR "water exercises":ti,ab,kw OR "water training":ti,ab,kw OR "pool-based":ti,ab,kw OR "pool based":ti,ab,kw OR "pool therapy":ti,ab,kw OR "pool exercise":ti,ab,kw OR "pool exercises":ti,ab,kw OR swimming:ti,ab,kw OR "swim training":ti,ab,kw OR "swimming program":ti,ab,kw OR "swimming programs":ti,ab,kw OR "swimming programme":ti,ab,kw OR "swimming programmes":ti,ab,kw OR Halliwick:ti,ab,kw OR "multisystem aquatic therapy":ti,ab,kw) AND (random*:ti,ab,kw OR randomly:ti,ab,kw OR trial:ti,ab,kw OR controlled:ti,ab,kw OR "control group":ti,ab,kw OR "control groups":ti,ab,kw OR assign*:ti,ab,kw OR allocat*:ti,ab,kw) |
| Web of Science | TS=(("autism spectrum disorder" OR "autism spectrum disorders" OR ASD OR autism OR autistic OR "autistic disorder" OR "autistic disorders" OR Asperger* OR "pervasive developmental disorder" OR "pervasive developmental disorders" OR PDD OR "PDD-NOS" OR "childhood autism") AND(hydrotherap* OR aquatic OR "aquatic therapy" OR "aquatic therapies" OR "aquatic exercise" OR "aquatic exercises" OR "aquatic intervention" OR "aquatic interventions" OR "aquatic program" OR "aquatic programs" OR "aquatic programme" OR "aquatic programmes" OR "aquatic training" OR "water-based" OR "water based" OR "water exercise" OR "water exercises" OR "water training" OR "pool-based" OR "pool based" OR "pool therapy" OR "pool exercise" OR "pool exercises" OR swimming OR "swim training" OR "swimming program" OR "swimming programs" OR "swimming programme" OR "swimming programmes" OR Halliwick OR "multisystem aquatic therapy")AND("randomized controlled trial" OR "randomized controlled trials" OR "randomised controlled trial" OR "randomised controlled trials" OR "controlled clinical trial" OR "controlled clinical trials" OR random* NEAR/3 trial* OR random* NEAR/3 control* OR randomly OR controlled OR "control group" OR "control groups" OR assign* OR allocat*)) |
| Scopus | TITLE-ABS-KEY(("autism spectrum disorder" OR "autism spectrum disorders" OR ASD OR autism OR autistic OR "autistic disorder" OR "autistic disorders" OR Asperger* OR "pervasive developmental disorder" OR "pervasive developmental disorders" OR PDD OR "PDD-NOS" OR "childhood autism") AND(hydrotherap* OR aquatic OR "aquatic therapy" OR "aquatic therapies" OR "aquatic exercise" OR "aquatic exercises" OR "aquatic intervention" OR "aquatic interventions" OR "aquatic program" OR "aquatic programs" OR "aquatic programme" OR "aquatic programmes" OR "aquatic training" OR "water-based" OR "water based" OR "water exercise" OR "water exercises" OR "water training" OR "pool-based" OR "pool based" OR "pool therapy" OR "pool exercise" OR "pool exercises" OR swimming OR "swim training" OR "swimming program" OR "swimming programs" OR "swimming programme" OR "swimming programmes" OR Halliwick OR "multisystem aquatic therapy") AND ("randomized controlled trial" OR "randomized controlled trials" OR "randomised controlled trial" OR "randomised controlled trials" OR "controlled clinical trial" OR "controlled clinical trials" OR random* W/3 trial* OR random* W/3 control* OR randomly OR controlled OR "control group" OR "control groups" OR assign* OR allocat*)) |
| EBSCOhost | ((TI ("autism spectrum disorder" OR "autism spectrum disorders" OR ASD OR autism OR autistic OR "autistic disorder" OR "autistic disorders" OR Asperger* OR "pervasive developmental disorder" OR "pervasive developmental disorders" OR PDD OR "PDD-NOS" OR "childhood autism") OR AB ("autism spectrum disorder" OR "autism spectrum disorders" OR ASD OR autism OR autistic OR "autistic disorder" OR "autistic disorders" OR Asperger* OR "pervasive developmental disorder" OR "pervasive developmental disorders" OR PDD OR "PDD-NOS" OR "childhood autism")) AND (TI (hydrotherap* OR aquatic OR "aquatic therapy" OR "aquatic therapies" OR "aquatic exercise" OR "aquatic exercises" OR "aquatic intervention" OR "aquatic interventions" OR "aquatic program" OR "aquatic programs" OR "aquatic programme" OR "aquatic programmes" OR "aquatic training" OR "water-based" OR "water based" OR "water exercise" OR "water exercises" OR "water training" OR "pool-based" OR "pool based" OR "pool therapy" OR "pool exercise" OR "pool exercises" OR swimming OR "swim training" OR "swimming program" OR "swimming programs" OR "swimming programme" OR "swimming programmes" OR Halliwick OR "multisystem aquatic therapy") OR AB (hydrotherap* OR aquatic OR "aquatic therapy" OR "aquatic therapies" OR "aquatic exercise" OR "aquatic exercises" OR "aquatic intervention" OR "aquatic interventions" OR "aquatic program" OR "aquatic programs" OR "aquatic programme" OR "aquatic programmes" OR "aquatic training" OR "water-based" OR "water based" OR "water exercise" OR "water exercises" OR "water training" OR "pool-based" OR "pool based" OR "pool therapy" OR "pool exercise" OR "pool exercises" OR swimming OR "swim training" OR "swimming program" OR "swimming programs" OR "swimming programme" OR "swimming programmes" OR Halliwick OR "multisystem aquatic therapy")) AND (TI ("randomized controlled trial" OR "randomized controlled trials" OR "randomised controlled trial" OR "randomised controlled trials" OR "controlled clinical trial" OR "controlled clinical trials" OR (random* N3 trial*) OR (random* N3 control*) OR randomly OR controlled OR "control group" OR "control groups" OR assign* OR allocat*) OR AB ("randomized controlled trial" OR "randomized controlled trials" OR "randomised controlled trial" OR "randomised controlled trials" OR "controlled clinical trial" OR "controlled clinical trials" OR (random* N3 trial*) OR (random* N3 control*) OR randomly OR controlled OR "control group" OR "control groups" OR assign* OR allocat*))) |

Table S3. Baseline characteristics and potential effect modifiers of included studies

| **Study/report** | **Country or region** | **Study design and sample size** | **Participant characteristics** | **Aquatic intervention** | **Comparator** | **Exercise protocol** | **Main outcomes** |
| --- | --- | --- | --- | --- | --- | --- | --- |
| **Adib 2019 / Adib 2024** | Iran | Randomized parallel or stratified randomized controlled trial; related reports from the same trial family | Boys; approximately 8–14 years old; Adib 2019: N = 20, aquatic exercise/control = 10/10; Adib 2024: N = 30, 10 participants per group | Structured aquatic training | Waiting control, maintenance of usual activities, or Kata training | 10 weeks, 2 sessions/week, 60 min/session | Stereotyped behavior; sleep |
| **Mills 2020** | Australia | Randomized crossover pilot trial; N = 8 | 8.72 ± 1.99 years; 6 boys/2 girls | Hydrotherapy/aquatic activity | Four-week control period within the same participants | 4 weeks, 1 session/week, 45 min/session | CBCL mental health and behavioral outcomes |
| **Albadry 2026** | Egypt | Assessor-blinded randomized parallel trial; randomized N = 53, completed N = 50; aquatic training/land exercise = 25/25 | 3–5 years old; boys and girls included | Aquatic training | Land-based exercise | 12 weeks, 3 sessions/week, 45 min/session | PDMS-2; CARS-2; PedsQL |
| **Koçak 2026** | Türkiye | Randomized parallel trial; randomized N = 40, completed N = 35; aquatic exercise/control = 17/18 | Boys; 8.4 ± 2.1 years | Adapted aquatic exercise | Usual education | 16 weeks, 2 sessions/week, 50 min/session | ABC; flexibility; handgrip strength |
| **Marzouki 2022** | Tunisia | Randomized three-arm parallel trial; N = 22; technical aquatic training/game-based aquatic training/control = 8/8/6 | Approximately 6.3–6.4 years; 20 boys/2 girls | Technical or game-based aquatic training | Usual physical activity | 8 weeks, 2 sessions/week, 50 min/session | Gross motor skills; emotion regulation; stereotyped behavior |
| **Liu 2026** | China | Pilot randomized parallel trial; N = 12; aquatic exercise/control = 6/6 | Boys; aquatic group 7.25 ± 0.41 years, control group 7.17 ± 0.40 years | Halliwick 10-point aquatic exercise program | No additional structured hydrotherapy | 12 weeks, 3 sessions/week, 60–70 min/session | CARS; ATEC; CSIS; balance; HAAR |
| **AdibSaber VitD 2024** | Iran | Randomized four-arm trial; N = 40, 10 participants per group | Boys; mean age 10.90 years, range 6–14 years | Aquatic exercise; aquatic exercise combined with vitamin D | Vitamin D, combined intervention, or no intervention | 10 weeks, 2 sessions/week, 60 min/session | IL-6; IL-10; social impairment |
| **Zhao 2024** | China | Randomized parallel trial; randomized N = 31, completed N = 30; aquatic exercise/control = 16/14 | Boys; aquatic group 10.69 ± 0.95 years, control group 10.79 ± 1.05 years | Moderate-intensity aquatic exercise | Supervised unstructured land-based free activity | 12 weeks, 4 sessions/week, 60–70 min/session | Inhibitory control; working memory; cognitive flexibility; BDNF |

Note: Adib 2019 and Adib 2024 were treated as related reports from the same trial family. When multiple reports or intervention arms were available, data were handled to avoid double-counting participants in the quantitative synthesis.

Table S4. Complete post-intervention value analyses and fixed-effect sensitivity analyses

| **Outcome** | **Model** | **k** | **g** | **CI lower** | **CI upper** | **p** | **Q** | **Q p** | **I²** | **tau²** |
| --- | --- | --- | --- | --- | --- | --- | --- | --- | --- | --- |
| overall_asd | Random-effects REML; KNHA | 6 | 1.314 | 0.634 | 1.995 | 0.004 | 9.005 | 0.109 | 45.482 | 0.178 |
| motor_function | Random-effects REML; KNHA | 4 | 2.345 | -0.113 | 4.803 | 0.056 | 37.830 | 0.000 | 90.206 | 2.188 |
| cars_severity | Random-effects REML; KNHA | 2 | 1.390 | 1.306 | 1.474 | 0.003 | 0.001 | 0.982 | 0.000 | 0.000 |
| cars_severity | Fixed-effect sensitivity | 2 | 1.390 | 0.828 | 1.953 | 0.000 | 0.001 | 0.982 | 0.000 | 0.000 |
| stereotypy | Random-effects REML; KNHA | 2 | 0.706 | -6.920 | 8.332 | 0.448 | 2.985 | 0.084 | 66.499 | 0.479 |
| stereotypy | Fixed-effect sensitivity | 2 | 0.687 | 0.006 | 1.367 | 0.048 | 2.985 | 0.084 | 66.499 | 0.000 |
| social_function | Random-effects REML; KNHA | 2 | 1.009 | -13.586 | 15.605 | 0.541 | 7.786 | 0.005 | 87.156 | 2.300 |
| social_function | Fixed-effect sensitivity | 2 | 0.996 | 0.189 | 1.803 | 0.016 | 7.786 | 0.005 | 87.156 | 0.000 |

Table S5. Comparison of post-intervention value analyses and change-score sensitivity analyses

| ***Outcome*** | ***Scenario*** | ***k*** | ***g*** | ***CI lower*** | ***CI upper*** | ***p*** | ***I²*** | ***tau²*** |
| --- | --- | --- | --- | --- | --- | --- | --- | --- |
| overall_asd | Posttest main | 6 | 1.314 | 0.634 | 1.995 | 0.004 | 45.482 | 0.178 |
| motor_function | Posttest main | 4 | 2.345 | -0.113 | 4.803 | 0.056 | 90.206 | 2.188 |
| cars_severity | Posttest main | 2 | 1.390 | 1.306 | 1.474 | 0.003 | 0.000 | 0.000 |
| stereotypy | Posttest main | 2 | 0.706 | -6.920 | 8.332 | 0.448 | 66.499 | 0.479 |
| social_function | Posttest main | 2 | 1.009 | -13.586 | 15.605 | 0.541 | 87.156 | 2.300 |
| overall_asd | Change Morris d_ppc2; r=0.5 | 6 | 1.267 | 0.314 | 2.219 | 0.019 | 75.578 | 0.644 |
| motor_function | Change Morris d_ppc2; r=0.5 | 4 | 3.360 | -2.333 | 9.053 | 0.157 | 97.896 | 10.685 |
| cars_severity | Change Morris d_ppc2; r=0.5 | 2 | 2.018 | -10.008 | 14.043 | 0.279 | 84.958 | 1.527 |
| stereotypy | Change Morris d_ppc2; r=0.5 | 2 | 0.638 | -3.490 | 4.766 | 0.300 | 0.000 | 0.000 |
| social_function | Change Morris d_ppc2; r=0.5 | 2 | 0.731 | -8.139 | 9.601 | 0.485 | 70.182 | 0.685 |
| overall_asd | Change Morris d_ppc2; r=0.3 | 6 | 1.283 | 0.318 | 2.248 | 0.019 | 69.262 | 0.622 |
| motor_function | Change Morris d_ppc2; r=0.3 | 4 | 3.367 | -2.349 | 9.083 | 0.158 | 97.353 | 10.707 |
| cars_severity | Change Morris d_ppc2; r=0.3 | 2 | 2.044 | -9.956 | 14.045 | 0.276 | 80.359 | 1.445 |
| stereotypy | Change Morris d_ppc2; r=0.3 | 2 | 0.643 | -3.484 | 4.769 | 0.298 | 0.000 | 0.000 |
| social_function | Change Morris d_ppc2; r=0.3 | 2 | 0.750 | -8.106 | 9.606 | 0.477 | 59.258 | 0.579 |
| overall_asd | Change Morris d_ppc2; r=0.7 | 6 | 1.249 | 0.310 | 2.188 | 0.019 | 82.747 | 0.667 |
| motor_function | Change Morris d_ppc2; r=0.7 | 4 | 3.352 | -2.319 | 9.024 | 0.157 | 98.476 | 10.672 |
| cars_severity | Change Morris d_ppc2; r=0.7 | 2 | 1.991 | -10.050 | 14.032 | 0.283 | 89.556 | 1.610 |
| stereotypy | Change Morris d_ppc2; r=0.7 | 2 | 0.630 | -3.499 | 4.758 | 0.303 | 33.100 | 0.070 |
| social_function | Change Morris d_ppc2; r=0.7 | 2 | 0.712 | -8.165 | 9.589 | 0.494 | 81.106 | 0.792 |

Table S6. Leave-one-out sensitivity analyses

| **Outcome** | **Excluded study** | **g** | **CI lower** | **CI upper** | **p** | **I²** | **tau²** |
| --- | --- | --- | --- | --- | --- | --- | --- |
| overall_asd | Albadry 2026 | 1.297 | 0.356 | 2.239 | 0.019 | 55.496 | 0.325 |
| overall_asd | Adibsaber 2024 | 1.193 | 0.460 | 1.925 | 0.011 | 41.968 | 0.143 |
| overall_asd | Adib 2019 | 1.316 | 0.408 | 2.224 | 0.016 | 58.117 | 0.290 |
| overall_asd | Kocak 2026 | 1.242 | 0.345 | 2.138 | 0.018 | 53.207 | 0.266 |
| overall_asd | Liu 2026 | 1.304 | 0.424 | 2.185 | 0.015 | 57.245 | 0.256 |
| overall_asd | Marzouki 2022 | 1.525 | 1.174 | 1.876 | 0.000 | 0.000 | 0.000 |
| motor_function | Albadry 2026 | 1.600 | -0.706 | 3.907 | 0.096 | 74.257 | 0.693 |
| motor_function | Kocak 2026 | 2.972 | -0.149 | 6.093 | 0.055 | 78.905 | 1.266 |
| motor_function | Liu 2026 | 2.398 | -2.230 | 7.026 | 0.156 | 94.252 | 3.273 |
| motor_function | Marzouki 2022 | 2.398 | -2.275 | 7.071 | 0.158 | 93.163 | 3.343 |

Table S7. Sensitivity analysis for within-study correlation coefficients in the motor function outcome

| **rho** | **k** | **g** | **CI lower** | **CI upper** | **p** | **I²** | **tau²** |
| --- | --- | --- | --- | --- | --- | --- | --- |
| 0.500 | 4 | 2.345 | -0.113 | 4.803 | 0.056 | 90.206 | 2.188 |
| 0.300 | 4 | 2.476 | 0.013 | 4.938 | 0.049 | 91.198 | 2.212 |
| 0.700 | 4 | 2.147 | -0.435 | 4.730 | 0.077 | 90.500 | 2.393 |

Table S8. Sensitivity analysis for the motor function outcome after excluding estimated Albadry data

| **Scenario** | **k** | **g** | **CI lower** | **CI upper** | **p** | **I²** | **tau²** |
| --- | --- | --- | --- | --- | --- | --- | --- |
| Posttest, excluding estimated Albadry data | 3 | 1.600 | -0.706 | 3.907 | 0.096 | 74.257 | 0.693 |
| Morris d_ppc2 r=0.5, excluding estimated Albadry data | 3 | 3.500 | -7.703 | 14.703 | 0.311 | 98.463 | 18.211 |

Table S9. Formal tests for subgroup differences

| **Outcome** | **Moderator** | **QM** | **df** | **P for difference** | **Status** |
| --- | --- | --- | --- | --- | --- |
| overall_asd | control_type | 2.435 | 1.000 | 0.194 | exploratory Knapp-Hartung meta-regression; total k=6, smallest subgroup k=2 |
| overall_asd | duration_group | 0.375 | 1.000 | 0.573 | exploratory Knapp-Hartung meta-regression; total k=6, smallest subgroup k=3 |
| overall_asd | frequency_group | 0.034 | 1.000 | 0.863 | exploratory Knapp-Hartung meta-regression; total k=6, smallest subgroup k=2 |
| motor_function | control_type | 2.164 | 1.000 | 0.279 | exploratory Knapp-Hartung meta-regression; total k=4, smallest subgroup k=2 |
| motor_function | duration_group |  |  |  | subgroup difference not tested: at least one subgroup has only 1 study |
| motor_function | frequency_group | 2.195 | 1.000 | 0.277 | exploratory Knapp-Hartung meta-regression; total k=4, smallest subgroup k=2 |

Table S10. Single-study outcomes summarized narratively

| **Outcome domain** | **Study** | **Available data** | **Reason for not pooling** |
| --- | --- | --- | --- |
| Sleep | Adib trial family | CSHQ total and subscales | One independent trial family after deduplication |
| Mental health / psychological behaviour | Mills 2020 | CBCL outcomes from a crossover design | Paired correlation and adequate crossover information unavailable; no independent-groups analysis |
| Emotion regulation | Marzouki 2022 | Emotion regulation and lability/negativity | One independent study |
| Sensory integration | Liu 2026 | CSIS subdomains at T2 | One independent study |
| Aquatic skills | Liu 2026 | HAAR subdomains at T2 | One independent study |
| Quality of life | Albadry 2026 | PedsQL | One independent study |
| Executive function | Zhao 2024 | Published MD and 95% CI; posttest means/SDs not numerically reported | One independent study; no chart-derived pseudo-precision |
| IL-6 | Adibsaber 2024 | Pure aquatic arm versus no-intervention control | One independent study |
| IL-10 | Adibsaber 2024 | Pure aquatic arm versus no-intervention control | One independent study |
| BDNF | Zhao 2024 | Published MD 0.759 (95% CI 0.441 to 1.077) | One independent study; posttest means/SDs not numerically reported |
